# Across Multiple Species, Phytochemical Diversity and Herbivore Diet Breadth Have Cascading Effects on Herbivore Immunity and Parasitism in a Tropical Model System

**DOI:** 10.3389/fpls.2018.00656

**Published:** 2018-06-11

**Authors:** Heather L. Slinn, Lora A. Richards, Lee A. Dyer, Paul J. Hurtado, Angela M. Smilanich

**Affiliations:** Department of Biology, University of Nevada, Reno, Reno, NV, United States

**Keywords:** tropics, *Piper*, tri-trophic interactions, phytochemical diversity, parasitism, diet breadth, chemodiversity

## Abstract

Terrestrial tri-trophic interactions account for a large part of biodiversity, with approximately 75% represented in plant–insect–parasitoid interactions. Herbivore diet breadth is an important factor mediating these tri-trophic interactions, as specialisation can influence how herbivore fitness is affected by plant traits. We investigated how phytochemistry, herbivore immunity, and herbivore diet breadth mediate plant–caterpillar–parasitoid interactions on the tropical plant genus *Piper* (Piperaceae) at La Selva Biological station in Costa Rica and at Yanayacu Biological Station in Ecuador. We collected larval stages of one *Piper* generalist species, *Quadrus cerealis*, (Lepidoptera: Hesperiidae) and 4 specialist species in the genus *Eois* (Lepidoptera: Geometridae) from 15 different species of *Piper*, reared them on host leaf material, and assayed phenoloxidase activity as a measure of potential larval immunity. We combined these data with parasitism and caterpillar species diet breadth calculated from a 19-year database, as well as established values of phytochemical diversity calculated for each plant species, in order to test specific hypotheses about how these variables are related. We found that phytochemical diversity was an important predictor for herbivore immunity, herbivore parasitism, and diet breadth for specialist caterpillars, but that the direction and magnitude of these relationships differed between sites. In Costa Rica, specialist herbivore immune function was negatively associated with the phytochemical diversity of the *Piper* host plants, and rates of parasitism decreased with higher immune function. The same was true for Ecuador with the exception that there was a positive association between immune function and phytochemical diversity. Furthermore, phytochemical diversity did not affect herbivore immunity and parasitism for the more generalised herbivore. Results also indicated that small differences in herbivore diet breadth are an important factor mediating herbivore immunity and parasitism success for *Eois* at both sites. These patterns contribute to a growing body of literature that demonstrate strong cascading effects of phytochemistry on higher trophic levels that are dependent on herbivore specialisation and that can vary in space and time. Investigating the interface between herbivore immunity, plant chemical defence, and parasitoids is an important facet of tri-trophic interactions that can help to explain the enormous amount of biodiversity found in the tropics.

## Introduction

Tri-trophic interactions are an important feature of biotic communities and contribute to the maintenance of biodiversity as well as mediate ecosystem processes (Price et al., 1980; Hunter and Price, 1992; Agrawal, 2000; Price, 2002; Whitham et al., 2006). For instance, terrestrial plant–insect–predator/parasitoid interactions may make up approximately three quarters of the diversity of multicellular organisms (Price, 2002). Ecologists have found that tri-trophic interactions can shape community parameters, such as species diversity, functional diversity, primary productivity, and consumer abundance (Hairston et al., 1960; Ives et al., 2005; Singer and Stireman, 2005; Crutsinger et al., 2006; Johnson, 2008; O’Connor et al., 2016). Many tri-trophic studies have focused on how primary producers affect biotic communities through effects on densities or population dynamics of herbivores, mutualists, and natural enemies (Crutsinger et al., 2006; Crawford et al., 2007; Barbour et al., 2015). Plant chemical defence is one of the most important components of these bottom-up effects, and there is a rich literature documenting how chemistry affects plant–insect interactions (Fraenkel, 1959; Ehrlich and Raven, 1964; Schoonhoven et al., 2005; Hunter, 2016), via both negative and positive physiological and behavioural effects on herbivores and natural enemies (Smilanich et al., 2016). One clear gap in our knowledge of how phytochemistry influences tri-trophic interactions is empirical data that consider the entire suite of plant secondary metabolites in a species instead of focusing on one or two major compounds (Richards et al., 2010, 2016; Smilanich et al., 2016). Given that herbivores are exposed to the full array of compounds during their larval development and as adults, significant consideration should be given to the diversity of secondary metabolites found in plants (Hay et al., 1994; Richards et al., 2015). Here, we use phytochemical diversity as a metric of plant defence to investigate the effects on herbivore performance as measured by immune strength, and whether effects on the immune response cascade to impact parasitism success (Smilanich et al., 2009b; Richards et al., 2015; Hansen et al., 2017).

Research on the role of herbivore immunity as a mediator of tri-trophic interactions has been expanding over the last decade (Bukovinszky et al., 2009; Smilanich et al., 2009a; Richards et al., 2012; Singer et al., 2014; Lampert and Bowers, 2015). However, the majority of this work has been performed in temperate systems (but see: Smilanich et al., 2009b; Smilanich and Dyer, 2012; Hansen et al., 2017), where plant chemistry is typically less diverse and compounds may be less toxic (Coley and Barone, 1996; Dyer and Coley, 2002). In general, increased concentrations or mixture complexities of plant chemical compounds have a detrimental impact on herbivore immunity (Haviola et al., 2007; Smilanich et al., 2009a; Richards et al., 2010, 2016; Lampert, 2012; Hansen et al., 2017), but these effects can differentially influence the success of predators and parasitoids (Dyer et al., 2004; Bukovinszky et al., 2009; Richards et al., 2015). For instance, specialist caterpillars (*Junonia coenia*: Nymphalidae) have a weakened immune response due to sequestering higher concentrations of secondary metabolites, and this has been termed the ‘vulnerable host hypothesis’ (Smilanich et al., 2009a; Lampert and Bowers, 2015). More generally, specialised herbivores should be better adapted to diverse mixtures of secondary metabolites in their specific host plants, which may also protect specialists from natural enemies (e.g., Dyer, 1995); however, the energetic costs that accompany sequestration may be toxic to immune cells or may lead to reallocation of resources away from immune functions, rendering specialists more susceptible to parasitism (Smilanich et al., 2009a). Chemically defended or immune-compromised specialists may provide a ‘safe haven’ for parasitoids because they are less likely to be attacked or consumed by other natural enemies, which tend to avoid toxic specialist hosts (Dyer, 1995). Indeed, generalists are often better protected than specialists against parasitoids (Dyer and Gentry, 1999). The vulnerable host and safe haven hypotheses suggest that phytochemically defended plants may host specialist herbivores that are immunocompromised and more likely to be attacked by parasitoids (Smilanich et al., 2009a; Lampert et al., 2010).

The effect of host plant chemistry on the immune response also depends on the physiological ecology of the organism: herbivores that utilise metabolically expensive strategies, such as detoxification or sequestration, to tolerate host plant chemistry may incur physiological costs to eating toxic diets and experience compromised immune systems (Smilanich et al., 2009a). For example, the immune response of *Eois nympha* and *Eois apyraria* (Geometridae) caterpillars was suppressed when feeding on *Piper cencocladum* (Piperaceae) compared to other *Piper* host plants, and *P. cenocladum* is more phytochemically diverse than other *Piper* host species (i.e., *Piper imperiale*) (Hansen et al., 2017). Furthermore, Richards et al. (2010) found that a mixture of plant secondary metabolites from a neotropical shrub in the genus *Piper* (Piperaceae) affected a naïve generalist noctuid caterpillar (*Spodoptera*) differently from adapted specialist geometrid caterpillars (*Eois*), with *Spodoptera* experiencing high mortality through direct toxicity, and indirect negative effects of chemistry on *Eois* via increased levels of parasitism. Increased parasitism associated with host plant toxicity is also consistent with the hypothesis that higher phytochemical diversity may weaken a caterpillar’s immune response, leading to increased parasitoid success. This hypothesised association is best tested when direct effects of chemistry on adult parasitoids are ruled out, which is the case in experiments where caterpillars are naturally exposed to parasitoids first in the field and then subsequently assigned to feeding treatments in the laboratory (e.g., Smilanich et al., 2009b; Richards et al., 2010; Hansen et al., 2017). Similarly, iridoid glycosides sequestered by buckeye caterpillars (*J. coenia*) negatively affected the efficacy of encapsulation by these specialists (Smilanich et al., 2009a) but did not affect this same (encapsulation) measure of the immune response in the generalist caterpillar, *Grammia incorrupta* (Erebidae: Arctiinae) (Smilanich et al., 2011). Overall, there is growing evidence that plant chemistry may mediate herbivore susceptibility to parasitoids via the herbivore’s immunity and the strength or direction of this relationship is dependent on the level of specialisation of the plant–herbivore interaction. While previous studies have included how diet breadth may affect the ecoimmunology of tri-trophic interactions, there are other axes of variation that are likely to be important for modifying this relationship, including biogeographical differences among sites. For example, plant chemistry and tri-trophic interactions vary across elevations (Rodríguez-Castañeda et al., 2016) and with rainfall intensity (Cunningham et al., 1999), thus the same herbivore species may be affected differently by host plant chemistry and parasitoids across elevational and precipitation gradients, due to differences in chemistry and in enemy communities (Rodríguez-Castañeda et al., 2016).

**FIGURE 1:**
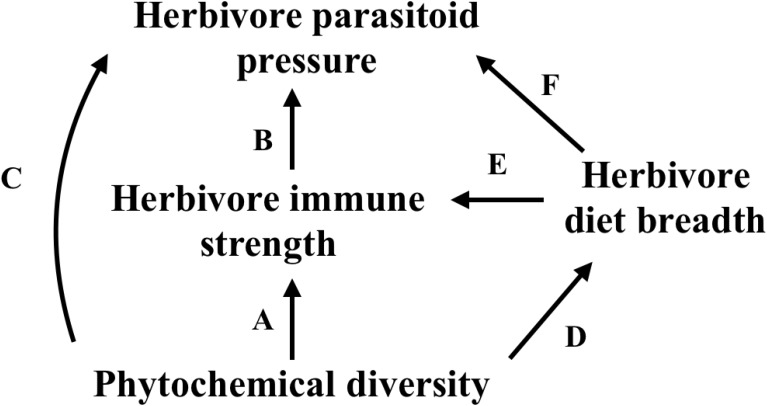
Meta-model that structured our *a priori* hypotheses. Letters over paths are associated with hypotheses in **Table 1**.

In this study, we used the tropical plant genus, *Piper*, the associated specialist herbivore genus, *Eois* (Lepidoptera: Geometridae), and a *Piper* generalist, *Quadrus cerealis* (Lepidoptera: Hesperiidae), to investigate whether variation in phytochemical diversity influences the strength of the herbivore immune response and associated levels of parasitism (**Figure 1** and **Table 1**). In addition to examining variation across these different herbivore species, we examined these relationships in two distinct ecosystems – a lowland wet forest in Costa Rica (La Selva, Sarapiqui) and a cloud forest in Ecuador (Yanayacu, Napo), which differ dramatically in temperature means and variance, annual rainfall, and elevation. Specifically, we designed our study to address the following questions: (1) How does phytochemical diversity influence herbivore immunity and levels of parasitism and how are these relationships affected by diet breadth? (2) How do these effects vary across different herbivore species and different locations?

**Table 1:** Description of the hypotheses and predictions behind each path in our supported SEM models.

Explanatory variables	Response variables	Paths	Hypotheses and predictions	References
Plant quality (phytochemical diversity)	Herbivore fitness (Immunity)	A	Plants with high phytochemical diversity are more likely to contain compounds that decrease herbivore fitness.	Berenbaum and Neal, 1985; Jones and Firn, 1991; Smilanich et al., 2009b; Diamond and Kingsolver, 2011; Lampert and Bowers, 2015
Herbivore fitness (immunity)	Herbivore parasitism	B	The immune system provides important protection against parasitoids, thus as the strength of the immune system decreases, parasitism increases.	Bukovinszky et al., 2009; Smilanich et al., 2009a; Quintero et al., 2014
Plant quality (phytochemical diversity)	Herbivore parasitism	C	Low plant quality caused by toxic secondary metabolites, and higher phytochemical diversity are more likely to weaken herbivores via the presence of bioactive compounds and/or toxic synergies, increasing parasitoid success.	Lill et al., 2002; Bukovinszky et al., 2009; Richards et al., 2010; Sternberg et al., 2012; Hunter, 2016
Plant quality (phytochemical diversity)	Herbivore diet breadth	D	Plants with greater diversity of phytochemical compounds are more likely to host specialised herbivores that have adapted to bioactive compounds and/or toxic synergies.	Becerra, 2007, 2015; Dyer et al., 2003, 2007; Richards et al., 2015
Herbivore diet breadth	Herbivore fitness (immunity)	E	Specialist herbivores are adapted to detoxifying or sequestering toxic plant compounds and will perform better on their host plants than generalists.	Coley et al., 2006; Richards et al., 2010; Lampert, 2012
Herbivore diet breadth	Herbivore parasitism	F	Herbivores that feed on a greater number of plants are exposed to a greater variety of toxic plant compounds which weaken herbivores, increasing parasitoid success.	Barbosa et al., 1991; Carvalheiro et al., 2010; Lampert et al., 2011; Reudler et al., 2011

## Materials and Methods

### Study Sites

Our study took place at two different field stations in the neotropics: (1) La Selva Biological Station, Heredia Province, Costa Rica (10° 26′ N 83° 59′ W) and (2) Yanayacu Biological station, Napo Province, Ecuador (00° 36′ S 77° 53′ W). The La Selva Biological reserve is 1600 ha of lowland rainforest and ranges from 35 to 140 m in elevation and is surrounded by a combination of disturbed, agricultural habitat, and natural forest. The mean annual precipitation is approximately 4200 mm. Sampling at Yanayacu Biological Station included the 100 ha owned by the station as well as thousands of hectares of surrounding cloud forest on the slopes of the eastern Andes. The elevation at the station is 2100 m and the annual precipitation is approximately 2624 mm.

### *Piper*–*Eois*, *Piper*–*Quadrus* System

The plant genus *Piper* (Piperaceae) is an emerging tropical model system for studying tri-trophic interactions because of the growing knowledge on its evolutionary history, genomics, plant chemistry, distribution, and insect communities (Marquis, 1991; Greig, 1993; Dyer and Palmer, 2004; Richards et al., 2015; Glassmire et al., 2016; Salazar et al., 2016). Currently there are over 2000 species of *Piper* that have been identified pantropically, with approximately 1300 species occurring in the neotropics, 50 species present at the La Selva Biological station and 20 present at the Yanayacu station. *Piper* is a phytochemically diverse genus, including compounds from at least 15 classes, and a total of 667 individual compounds have been discovered (Richards et al., 2016). In this study, we used previously published data quantifying phytochemical diversity for multiple *Piper* species (Richards et al., 2015). Each of the *Piper* species in this experiment had a fixed diversity value and therefore no intra-specific variation was quantified. Briefly, phytochemical diversity is an effective number of functional groups, transformed from a Simpson’s diversity entropy calculated from proton nuclear magnetic resonance (^1^H–NMR), which incorporates both mixture complexity and structurally complexity, the two key components of chemical diversity (Richards et al., 2015).

*Piper* species host diverse lepidopteran herbivore communities that vary in diet breadth (Dyer and Palmer, 2004). Caterpillars in the genus *Eois* (Lepidoptera: Geometridae) are *Piper* specialists that feed exclusively on 1–4 different *Piper* species (Connahs et al., 2009). They are one of the most well studied and abundant genera of caterpillars found on *Piper*, and over 80% of *Eois* species are found in the neotropics with others in Africa, Asia, and Australia (Rodríguez-Castañeda et al., 2010; Brehm et al., 2011). In contrast, the *Piper* skipper, *Q. cerealis* (Lepidoptera: Hesperiidae), has been recorded feeding on 23 *Piper* species; in this paper we categorise this skipper as a *Piper* generalist^1^ (Dyer et al., 2010).

### Long Term Rearing Databases

Since 1991, principal investigators, students, volunteers, and technicians have been collecting plant–herbivore–parasitism interaction data in Costa Rica (e.g., from Dyer and Floyd, 1993; Hansen et al., 2017). We used data from 1996 to 2015 in this database (these years included the most complete parasitism data) to determine simple taxonomic diet breadth for herbivores (number of host plants documented for a caterpillar species) and parasitism frequency, quantified as the total number of parasitized caterpillars divided by the total number of caterpillars reared to adult plus parasitoid (parasitoids)/(healthy adults + parasitoids) (Gentry and Dyer, 2002; Hansen et al., 2017; **Tables 2**, **3**). Data consisted mainly of entries from La Selva Biological Station, but also from other areas nearby such as Braulio Carrillo National Park and the Tirimbina Biological Reserve. Primarily third instar caterpillars were collected year-round in all forest types and reared on the host plant from which they were collected in ambient conditions until they pupated and eclosed into adulthood, or if parasitized prior to collection, until they succumbed to parasitism. Data were collected on the caterpillar species, the host plant it was found on, and whether it reached adulthood or was parasitized (for detailed methods see Gentry and Dyer, 2002). Using these data, we evaluated herbivore immunity for four different *Eois* species collected from five different *Piper* species (**Table 2**). For these species, we found a total of 2011 records in our database with 900 caterpillars successfully reaching adulthood (**Table 2**). Additionally, we collected *Q. cerealis* from 10 different *Piper* species, though we have records of larvae feeding on 23 different *Piper* species (**Table 3**). We recorded 117 instances of *Q. cerealis* on these 10 *Piper* species with 75 caterpillars successfully reaching adulthood (**Table 3**). **Tables 2**, **3** summarise the sample sizes of larvae collected for immune assays and long-term parasitism sample sizes for those same species.

**Table 2:** *Eois* caterpillars and their host plants collected for immune assays.

Site	*Eois* spp.	*Piper* spp.		Database		
			*n*	Records	Adults	% parasitized
Costa Rica	*Eois nympha*	*Piper biseriatum*	9	44	7	29
		*Piper cenocladum*	28	921	317	18
	*Eois apyraria*	*Piper cenocladum*	1	328	164	8.4
		*Piper imperiale*	7	616	359	1.4
	*Eois russearia*	*Piper sancti-felicis*	12	48	24	4
	*Eois mexicaria*	*Piper umbricola*	13	54	29	0

Total			70	2011	900	

Ecuador	Six black two pink spots	*Piper baezanum*	2	6	1	0
		*Piper kelleyi*	16	1792	700	14
		*Piper lancifolium*	1	1	0	0
	Lime slime	*Piper baezanum*	1	3	1	0
		*Piper kelleyi*	7	9	0	0
	Two black spots	*Piper kelleyi*	27	83	29	3.3
		*Piper lancifolium*	1	1	0	0
	*Eois viridiflava* Dognin	*Piper baezanum*	1	2	0	0
		*Piper lancifolium*	20	36	0	0
	Pink spots funk	*Piper kelleyi*	3	86	37	8.1
		*Piper lancifolium*	1	1	0	0
	Eight black blur	*Piper baezanum*	1	1	9	0
	*Eois beebei* Fletcher	*Piper kelleyi*	1	36	19	14
	*Eois ignefumataPdfLatex* Dognin	*Piper kelleyi*	1	22	13	19

Total			83	2079	809	

*Sample size of the immune assays is indicated by “*n*”. Host plant-caterpillar species information from two multi-year databases includes all collection records, specified in the ‘records’ column, caterpillars that made it to adulthood and parasitism percentage.*

**Table 3:** *Quadrus cerealis* caterpillars and their host plants collected for immune assays.

Site	*Piper* spp.		Database		
		*n*	Records	Adults	% parasitized
Costa Rica	*Piper arboreum*	3	2	2	0
	*Piper cenocladum*	1	4	3	25
	*Piper colonense*	13	16	13	38
	*Piper garagaranum*	1	3	2	33
	*Piper imperiale*	6	2	1	50
	*Piper multiplinervium*	19	26	26	7.7
	*Piper pseudobumbratum*	1	1	1	0
	*Piper reticulatum*	18	62	26	68
	*Piper trigonum*	2	0	0	0
	*Piper umbricola*	1	1	1	0
Total		65	117	75	

*Sample size of the immune assays is indicated by “*n*”. Host plant-caterpillar species information from a 19-year database includes all collection records, specified in the ‘records’ column, caterpillars that made it to adulthood and parasitism percentage.*

The same data collection procedure was utilised at the Ecuador site, where the database spans 15 years (2001–2015). Larvae were collected in the cloud forest surrounding Yanayacu Biological Station. At this site, we measured the immune response from eight *Eois* morphospecies feeding on three different *Piper* species (**Table 2**). We had 2079 records of our *Eois* morphospecies in our database with 809 caterpillars successfully reaching adulthood (**Table 2**). We calculated diet breadth and levels of parasitism using the same method at both sites. Diet breadth was calculated as the number of *Piper* species on which a caterpillar species was found feeding and successfully reared to adult moth or parasitoid. As with the Costa Rica data, parasitism frequency was calculated as the number of parasitism events for each caterpillar species divided by the total number of successfully reared adults + parasitoids.

### Immune Assay

Phenoloxidase (hereafter PO) is an important enzyme for triggering the melanization process, a mechanism of innate immunity involving deposition of pigments on foreign bodies (Beckage, 2008; González-Santoyo and Córdoba-Aguilar, 2012). It is typically stored in hemolymph cells in a non-activated form called prophenoloxidase (proPO) since active PO can have locally toxic effects (Cerenius et al., 2008). Upon infection or natural enemy attack, proPO is converted to the active form, PO, which catalyses the cascade to produce melanin. Phenoloxidase has been shown to be an important part of the immune response in arthropods, protecting them from bacteria, viruses, and parasitoids (Cerenius et al., 2008). We measured the activity of the PO enzyme as an indicator of the strength of the herbivore immune response (González-Santoyo and Córdoba-Aguilar, 2012). We collected four species of early instar caterpillars from five different plant species and reared them on the host plant in which they were found in ambient conditions until they reached 5th instar. To measure PO activity (modified from Adamo, 2004), we took 2 μL of hemolymph from each *Eois* caterpillar (Costa Rica: *N* = 70, Ecuador: *N* = 83) and 5 μL from each *Q. cerealis* caterpillar (Costa Rica: *N* = 65), collected by puncturing the caterpillar with a pin and extracting hemolymph with a pipette. The volume of hemolymph was divided the into two Eppendorf tubes—one for cell-free PO found in the hemolymph at the time the hemolymph is taken (standing PO), and one for cell-bound PO, which is artificially activated by adding a chemical activator (total PO). The aliquots of hemolymph were added to 50 μl of phosphate buffered saline for *Eois* individuals and 100 μl PBS for *Q. cerealis* individuals. For the total PO in both species, 35 μl of chymotrypsin (1mg/mL) was added to the PBS-bound hemolymph, vortexed for 2 s, then incubated at room temperature for 20 min. During incubation, the substrate, dopamine, (0.0284 g/10 mL distilled water) was prepared. Since this compound is light sensitive, fresh dopamine was prepared daily. For *Eois*, we added 300 μl of dopamine to each Eppendorf tube, vortexed for 2 s, then added 25 μl of the dopamine-hemolymph mixture to a well plate. For *Q. cerealis*, we added 500 μl of dopamine to each Eppendorf tube, vortexed for 2 s, then added 200 μl of the dopamine-hemolymph mixture to a well plate. We used a spectrophotometer (BIO-RAD: iMark Microplate Absorbance Reader) at a wavelength of 490 nm to measure the activity of PO every 30 s for 45 min. We measured the slope, which was the rate of reaction, from the first 10 min because it was a linear increase. PO assays were performed in Costa Rica from January 2013 to December 2015 and in Ecuador from December 2015 to January 2016.

### Statistical Analyses

We used structural equation models (SEM) to evaluate 7 *a priori* hypotheses, which tested for bottom-up effects of phytochemical diversity and herbivore diet breadth on herbivore immunity and parasitism success (**Figure 1** and **Table 1**). We used the global estimation method in the R packages piecewiseSEM v.1.2.1 (Lefcheck, 2016) and lavaan v.0.5–23 to run our SEMs (Rosseel et al., 2017) in R v3.4.2 (R Core Team, 2017). We were not able to normalise the residuals of our data, so we chose a more robust estimator to account for non-normality and unequal variance instead of the default maximum likelihood method; this method is based on the Satterthwaite approach and is called the maximum likelihood estimation with robust standard errors and a mean and variance adjusted test statistic (Rosseel et al., 2017). Lastly, we used the same 7 hypotheses in our Ecuador dataset as we had no reason to believe that our systems should operate differently (**Figure 1** and **Table 1**).

For each site, we used a Bayesian mixed linear model to examine effects of phytochemical diversity on immune response. This approach allowed us to incorporate prior distributions from earlier studies using the same methodology (Smilanich and Dyer, 2012), also to account for Type II error (i.e., reporting actual probabilities of null hypotheses) and to test the generalizability of our results. Caterpillar species were a random effect in the model. Priors were generated from *E. nympha* and *E. apyraria* caterpillars collected on *P. biseriatum, P. cenocladum, P. imperiale*, and *P. urostachyum* at La Selva Biological Station in Costa Rica. The Bayesian model was estimated using SAS 9.4 (v13.1) procedure MCMC. We chose the quasi-Newton algorithm, convergence was assessed via visual examination of the trace plot, and the first 2,000 (burn-in) out of 10,000 samples were discarded, yielding robust posterior distributions for parameters. We report the posterior distributions of *B_1_* parameter estimates from this model for the effects of phytochemical diversity on rate of total PO absorbance per minute.

## Results

### Summary Statistics

Average immune response for *Eois*, as measured by total PO absorbance per minute (ΔAbs), was approximately equal across sites (*Eois:* Costa Rica: 0.03 ± 0.004 ΔAbs; Ecuador: 0.02 ± 0.001 ΔAbs; here and elsewhere, error is 1 SEM), and between specialist *Eois* and *Piper* generalist, *Q. cerealis* (*Q. cerealis*: 0.02 ± 0.002 ΔAbs). However, average parasitism level was higher for *Q. cerealis* (0.34 ± 0.03 parasitism frequency) compared to *Eois* at both sites (Costa Rica: 0.12 ± 0.01 parasitism frequency; Ecuador: 0.04 ± 0.01 parasitism frequency). Parasitoid families attacking the caterpillars also differed between sites and species. *Q. cerealis* parasitism was entirely tachinid parasitoids, while *Eois* parasitism in Costa Rica was 80% braconids, 8% tachinids, and 12% parasitism by other families. *Eois* parasitism in Ecuador was 24% tachinids, 41% braconids, and 35% parasitism by other families. Increases in phytochemical diversity had negative effects on the immune response in both Costa Rica and Ecuador, with posterior distributions of parameter estimates (from the mixed Bayesian model) similar to those reported previously for effects of diet on immune response. The negative effects of phytochemical diversity on immune response yielded parameter estimates at both sites that did not include a slope of zero; the combined mean slope for effect of NMR bin diversity on ΔAbs was -0.46.

### Structural Equation Models

**FIGURE 2:**
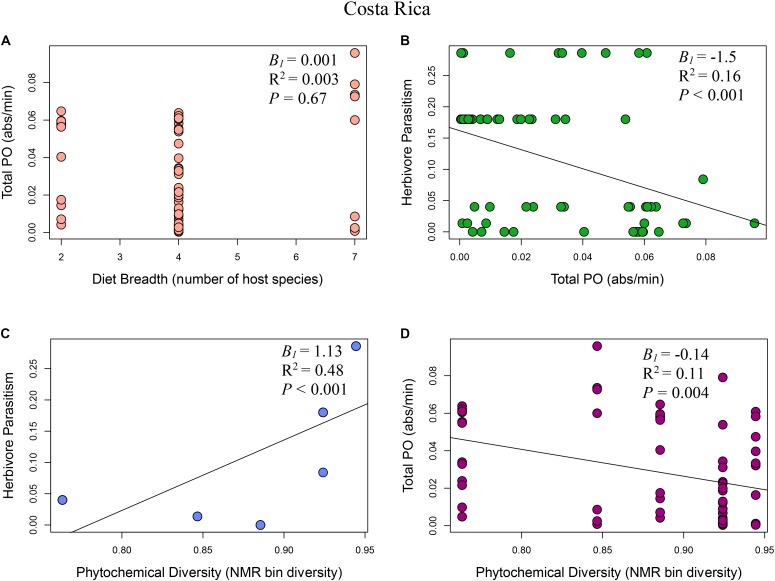
Multi-panel regression plots of *Eois* ecoimmunological parameters in Costa Rica: **(A)** Relationship between diet breadth, measured as number of host species, and *Eois* immune response, measured as total phenoloxidase absorbance per minute (*B_1_* = 0.001, *R*^2^ = 0.003, *F*_1,68_ = 0.18, *P* = 0.67). **(B)** *Eois* immune response and percent *Eois* parasitism (*B_1_* = –1.5, *R*^2^ = 0.16, *F*_1,68_ = 12.95, *P* < 0.001). **(C)** Phytochemical diversity, measured as NMR binned peak diversity, and *Eois* percent parasitism (*B_1_* = 1.13, *R*^2^ = 0.48, *F*_1,68_ = 63.78, *P* < 0.001). **(D)** Phytochemical diversity and *Eois* immune response (*B_1_* = –0.14, *R*^2^ = 0.11, *F*_1,68_ = 8.67, *P* = 0.004).

**FIGURE 3:**
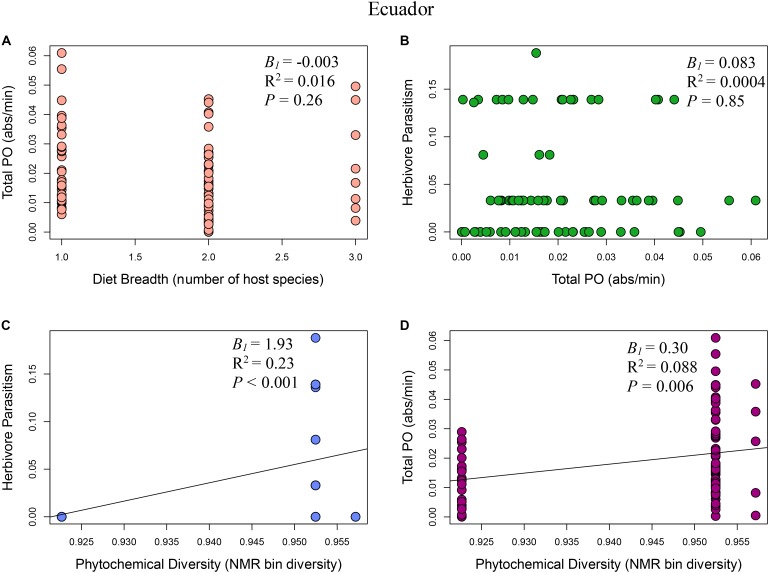
Multi-panel regression plots of *Eois* ecoimmunological parameters in Ecuador: **(A)** Relationship between diet breadth, measured as number of host species, and *Eois* immune response, measured as total phenoloxidase absorbance per minute (*B_1_* = –0.003, *R*^2^ = 0.016, *F*_1,81_ = 1.28, *P* = 0.26). **(B)** *Eois* immune response and percent *Eois* parasitism (*B_1_* = 0.083, *R*^2^ = 0.0004, *F*_1,81_ = 0.036, *P* = 0.85). **(C)** Phytochemical diversity, measured as NMR binned peak diversity, and *Eois* percent parasitism (*B_1_* = 1.93, *R*^2^ = 0.23, *F*_1,81_ = 23.82, *P* < 0.001). **(D)** Phytochemical diversity and *Eois* immune response (*B_1_* = 0.30, *R*^2^ = 0.088, *F*_1,81_ = 7.82, *P* = 0.006).

Overall, the best fit structural equation models supported the hypotheses that both phytochemical diversity and herbivore diet breadth are important factors shaping herbivore immunity and parasitism for *Eois* species in both Ecuador and Costa Rica, however, for some relationships, the directions of the effects were reversed from one site to another (**Table 4** and **Figures 2**, **3**). Tests of seven a priori models to explain the relationships of our measured variables were completed for both sites. In addition, we tested our models by bootstrapping missing data to even out sampling effort (see Supplementary Tables S1, S2). This analysis yielded only one of the same models as our initial analysis without the bootstrapped data for Costa Rica but not Ecuador (Model II, the diet breadth regulation hypothesis; Supplementary Table S3).

**Table 4:** Structural equation model (SEM) results from Costa Rica *Eois* and *Q. cerealis* study systems.

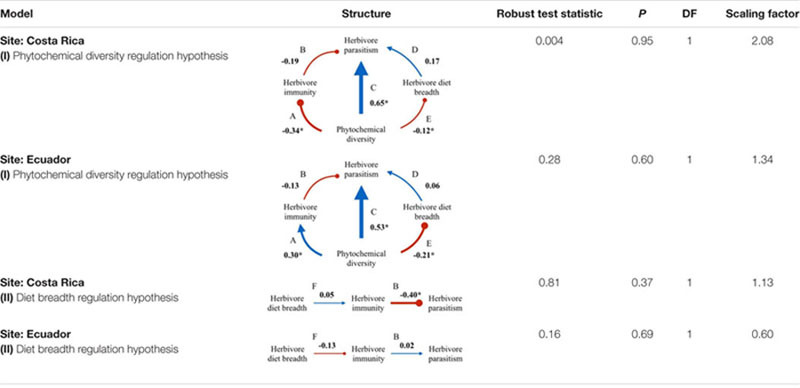

*Our hypotheses tested for: (I) ‘Phytochemical diversity regulation hypothesis’ – Phytochemical diversity having direct and indirect effects on higher trophic levels and which are mediated by both herbivore immunity and herbivore diet breadth (model fit: Robust test statistic = 0.004, df = 1, P = 0.95, scaling factor = 2.08), (II) ‘Diet breadth regulation hypothesis’ – Herbivore diet breadth is the main driver of herbivore immunity which in turn influences herbivore parasitism (model fit: Robust test statistic = 0.81, df = 1, P = 0.37, scaling factor = 1.13). SEM results from Ecuador Eois system. Our hypotheses tested for: (I) ‘Phytochemical diversity regulation hypothesis’ – Phytochemical diversity having direct and indirect effects on higher trophic levels and which are mediated by both herbivore immunity and herbivore diet breadth (model fit: Robust test statistic = 0.28, df = 1, P = 0.60, scaling factor = 1.34), (II) ‘Diet breadth regulation hypothesis’ – Herbivore diet breadth is the main driver of herbivore immunity which in turn influences herbivore parasitism (model fit: Robust test statistic = 0.16, df = 1, P = 0.69, scaling factor = 0.60). Asterisks represent significant path coefficients (P < 0.05).*

### Model I: Phytochemical Diversity Regulation Hypothesis

The phytochemical diversity regulation hypothesis (Model I) for our Costa Rica *Eois* data included phytochemical diversity as an exogenous variable with direct paths to herbivore immunity, herbivore diet breadth, and herbivore parasitism; the model also included effects of herbivore immunity and diet breadth on herbivore parasitism (Costa Rica model fit: Robust test statistic = 0.004, df = 1, *P* = 0.95, scaling factor = 2.08). This model supported the hypothesis that there is a strong direct positive effect of phytochemical diversity on herbivore parasitism (**Figure 2C**, standardised path coefficient (hereafter, spc) = 0.65, *P* < 0.01, slope (*B_1_*) = 1.13), showing that herbivores feeding on plants with high phytochemical diversity had higher parasitism rates. This model also showed that phytochemical diversity decreases herbivore immunity (**Figure 2D**, spc = -0.34, *P* < 0.01, *B_1_* = -0.14). It supports the hypothesis that higher herbivore immunity decreases herbivore parasitism frequency (**Figure 2B**, spc = -0.19, *P* = 0.08, *B_1_* = -1.52). Lastly, this model shows a negative effect of phytochemical diversity on herbivore diet breadth (i.e., *Piper* species with greater phytochemical diversity are consumed by more specialised *Eois* species; spc = -0.12, *P* = 0.03, *B_1_* = -2.66). In turn, herbivore diet breadth has a weak, positive effect on herbivore parasitism (i.e., generalists have higher levels of parasitism; spc = 0.17, *P* = 0.11, *B_1_* = 0.001). The same model was strongly supported by our Ecuador *Eois* data, however, the directions of some of the relationships were reversed (Ecuador model fit: Robust test statistic = 0.28, df = 1, *P* = 0.60, scaling factor = 1.34). Consistent with the Costa Rica data, phytochemical diversity has a strong positive effect on herbivore parasitism (**Figure 3C**, spc = 0.53, *P* < 0.01, *B_1_* = 1.93), however, in contrast to the Costa Rica data, phytochemical diversity has a positive effect on herbivore immunity (**Figure 3D**, spc = 0.30, *P* < 0.01, *B_1_* = 0.30). Phytochemical diversity has a negative effect on herbivore diet breadth (spc = -0.21, *P* < 0.01, *B_1_* = -9.55), and herbivore immunity negatively affects herbivore parasitism (**Figure 3B**, spc = -0.13, *P* = 0.22, *B_1_* = 0.08). Lastly, diet breath has no effect on herbivore parasitism (spc = 0.06, *P* = 0.62, *B_1_* = -0.003). Models for *Q. cerealis* caterpillars in Costa Rica did not fit the data, for example, a model where phytochemical diversity affects herbivore immunity, which in turn influences herbivore parasitism, was a poor fit to the data (model fit: Robust test statistic = 28.37, df = 1, *P* < 0.01, scaling factor = 0.50). However, a separate regression analysis showed that phytochemical diversity had a negative relationship with *Q. cerealis* parasitism [*B_1_* = -4.39, *F*_(1,63)_ = 15.25, *P* < 0.01].

### Model II: Diet Breadth Regulation Hypothesis

The diet breadth regulation hypothesis (model II) is a simpler model focusing on the effects of diet breadth on herbivore immunity and parasitism (Costa Rica model fit: Robust test statistic = 0.81, df = 1, *P* = 0.37, scaling factor = 1.13). In Costa Rica, this model shows that greater diet breadth (measured as *Eois* species that are documented feeding on a greater number of host plants) had a weak positive effect on herbivore immune response (**Figure 2A**, spc = 0.05, *P* = 0.76, *B_1_* = 0.001) and that immune function reduces parasitism success (**Figure 2B**, spc = -0.40, *P* < 0.01, *B_1_* = -1.52). The diet breadth regulation hypothesis was again supported by our Ecuador data (model II) (Ecuador model fit: Robust test statistic = 0.16, df = 1, *P* = 0.69, scaling factor = 0.60), but for this site, a greater diet breadth had a weak negative association with herbivore immunity (**Figure 3A**, spc = -0.13, *P* = 0.30, *B_1_* = -0.003), and herbivore immunity has no effect on herbivore parasitism (**Figure 3B**, spc = 0.02, *P* = 0.84, *B_1_* = 0.08).

## Discussion

Our results corroborate many other studies demonstrating that the chemistry of herbivore host plants, as well as herbivore diet breadth have strong effects on multiple aspects of herbivore ecology (Berenbaum and Neal, 1985; Haviola et al., 2007; Diamond and Kingsolver, 2011; Lampert and Bowers, 2015), including immunity and parasitism (Smilanich et al., 2009a; Hansen et al., 2017). A focus on the immune response allows for investigation of an important physiological parameter that is directly linked to protection against natural enemies (Smilanich et al., 2009b), putting our results in a strong tri-tropic context. It is also interesting that the relationships between phytochemical diversity, immunity, and parasitism were dependent upon the diet breadth of the specialist herbivores and that relationships varied across herbivore taxa and site. In Costa Rica, *Eois* feeding on *Piper* species with high phytochemical diversity had a weakened immune response, while the immune response of *Q. cerealis* was unaffected. It is important to note that the sample size for some herbivore species in Ecuador was small, which weakens the strength of our results (**Table 2**). For instance, three different herbivore species were only collected once (**Table 2**) – collecting many replicate herbivore species in the tropics can be difficult, depending on their abundance and distribution. Nevertheless, our results correspond with the importance of diet breadth in other results with this system (Richards et al., 2015). *Eois* data in Ecuador fit the same two models as in Costa Rica, however, some relationships were reversed. For example, in Costa Rica, individuals with a strong immune response had lower parasitism frequency (model II), however, in Ecuador herbivore immunity had almost no effect on parasitism frequency. This difference may be due to the differences in parasitoid pressure between the two sites. Compared to Ecuador, the database shows that *Eois* in Costa Rica have three times more parasitism by a relatively more specialised parasitoid community (Braconidae). Our Ecuador data include plant-caterpillar species pairs that are not well represented in our historical database and which have 0% parasitism as a result. We ruled out that this was driving our observed patterns by re-running our SEMs without plant-caterpillar species pairs that had low representation in our database, but found the same qualitative result. We therefore included these data points in our final analysis. Other possible particulars of the taxa and sites used for our study, such as degree of specialisation and elevation of the site, may also be responsible for these differences, but greater insight into those variables will require further experimentation using carefully selected taxa and locations.

Untangling relationships between plant chemistry, herbivores, and natural enemies has been a focus of insect ecology for decades (Price et al., 1980; Bernays and Graham, 1988; Dyer, 1995, 2011) and our results with *Eois* in Costa Rica are consistent with emerging paradigms of the importance of phytochemistry in mediating multi-trophic interactions. Most notably, we provide further support for the ‘safe haven hypothesis’ (Lampert et al., 2010) and the ‘vulnerable host hypothesis’ (Smilanich et al., 2009a). *Eois* data from Costa Rica support all aspects of this ‘safe haven hypothesis’ and data from both sites support the more general concept that changes in chemistry are likely to alter herbivore immunity and parasitism – the positive effects of phytochemical diversity on herbivore immunity in Ecuador are not inconsistent with this hypothesis, and they simply require further investigation to determine mechanisms causing this relationship. Furthermore, both SEM models (**Table 4**, Hypotheses I and II) are consistent with the growing body of evidence that the ability of an insect herbivore to mount an immune response is negatively associated with herbivore parasitism (Bukovinszky et al., 2009; Quintero et al., 2014), which is an important component of the ‘safe haven hypothesis,’ and some have argued that this is the best predictor of parasitism (Smilanich et al., 2009b; Greeney et al., 2012).

Other studies that support the ‘safe haven hypothesis’ (Gentry and Dyer, 2002; Lampert et al., 2010) or related hypotheses (i.e., ‘nasty host hypothesis’ Barbosa et al., 1991; Gauld et al., 1992) have focused on detoxification or sequestration of individual compounds or entire classes of compounds and have measured relative concentrations of those compounds (e.g., Haviola et al., 2007; Smilanich et al., 2009a; Lampert and Bowers, 2015). We utilise a different approach and consider the fact that phytochemical mixtures are complex and herbivores may be as susceptible to mixture complexity, synergies, or additive effects rather than just increases in concentrations of individual compounds or classes, such as tannins (Richards et al., 2015). One shortcoming of this approach is that results will require further investigation to get at mechanism. In Costa Rica, the immune responses of *Eois* species were negatively affected by increases in phytochemical diversity (**Table 4**, Hypothesis I). Another study with *Eois* on *Piper* found that changes in mixture complexity are associated with synergistic effects on parasitoid success (Richards et al., 2010). It is possible that host plants with higher phytochemical diversity are more likely to have synergistic effects on herbivores, impairing immune function, regardless of whether the mixtures are sequestered.

It is interesting to note that the results depended on taxon (*Quadrus* versus *Eois*) and site (Ecuador versus Costa Rica). Such variation is expected, and it is worth further investigation to determine conditions that are favourable for these chemically mediated tri-tropic interactions. Site and taxon were treated as random effects in the broader sense and were not statistically compared; nevertheless, it is interesting to consider possibilities for some of the differences across the two taxa and the two sites. Specialist *Eois* caterpillars in Costa Rica support our predictions, whereas, the same genus of caterpillars in Ecuador do not support any of our a priori models. Elevation is one clear difference between these sites, with the cloud forest in Ecuador situated 2,000 m higher than the lowland forest in Costa Rica. It is well known that herbivore development rates, herbivory, levels of predation, and herbivore diversity are lower at higher elevations, while parasitism and parasitoid diversity increase with elevation (Rodríguez-Castañeda et al., 2011, 2016), so it is not surprising that the specifics of chemically mediated tri-tropic interactions would vary with elevation. Reasons for the positive effect of phytochemical diversity on immunity at higher elevation are not obvious, but given the higher levels of parasitism and slow development rates, it is possible that maximised immunity is enhanced with slow development rates since larvae are exposed to parasitoids for longer periods of time. Similarly, there are many differences between the geometrid and hesperiid caterpillars utilised in our study, including diet breadth; however, one large difference is that *Quadrus* is a concealed feeder, and concealed feeders are affected less by phytochemical defence (Sandberg and Berenbaum, 1989; Berenbaum, 1990) and experience very high levels of parasitism (Gentry and Dyer, 2002). As such, *Q. cerealis* appeared to be unaffected by changes in chemistry and experienced extremely high levels of parasitism. There are likely unmeasured variables that influence immunity of hesperiids and more generally of concealed feeders, and it is certainly possible that the greater diet breadth played a role in the differences noted here.

In summary, our research builds on previous work investigating the effects of phytochemical diversity and herbivore diet breadth on ecoimmunology and tri-trophic interactions. These results support the hypothesis that variation in phytochemical diversity, rather than individual compounds, was a predictor of tri-trophic interactions and herbivore immunity (Richards et al., 2010, 2016). These patterns are also particularly important for understanding tropical systems, which are typically characterised by intense biotic interactions and high levels of diversity (Dyer and Coley, 2002; Novotny et al., 2006). Future work should investigate how much intraspecific phytochemical variation exists within these *Piper* species, how intraspecific variation compares across different *Piper* species, and what is driving that variation. Further, how does this intraspecific variation affect higher trophic levels and what are the differences in responses between predators and parasitoids. A field experiment evaluating the susceptibility of herbivores to parasitoids feeding on *Piper* of varying phytochemical profiles would greatly add to our understanding of the consequences of phytochemical diversity on herbivore immunity. Lastly, as the effects of global change worsen, including loss of tropical forests, the diversity of plant secondary metabolites is predicted to decrease, and understanding this diversity is a key part of documenting these losses.

## Author Contributions

LR, AS, and LD designed the experiments. HS performed the field work. HS and LD analysed the data. LD, LR, AS, and PH provided advice for the data analysis. HS wrote the first drafts. All authors contributed to additional draft of the manuscript.

## Conflict of Interest Statement

The authors declare that the research was conducted in the absence of any commercial or financial relationships that could be construed as a potential conflict of interest.

## Footnotes

1 http://www.caterpillars.org
