# Supplementary material for: Across Multiple Species, Phytochemical Diversity and Herbivore Diet Breadth Have Cascading Effects on Herbivore Immunity and Parasitism in a Tropical Model System
Source: Front Plant Sci. 2018 Jun 11;9:656. doi: 10.3389/fpls.2018.00656 (PMC6004389; doi:10.3389/fpls.2018.00656)
Supplement: Supplementary file 1 [file Table_1.docx]

***Supplementary Material***

**Across multiple species, phytochemical diversity and herbivore diet breadth have cascading effects on herbivore immunity and parasitism in a tropical model system**

**Heather L. Slinn^*^, Lora A. Richards, Lee A. Dyer, Paul J. Hurtado, and Angela M. Smilanich**

***Correspondence:** heather.slinn@gmail.com

**Supplementary Table 1.** Bootstrapping was used to impute missing data to account for differences in sampling effort. Data was bootstrapped to meet the sample size of the largest plant–caterpillar species pairs from our immune assays. We bootstrapped our data using the Hmisc v.4.0–3 (Harrell, 2015) package in R v3.4.2 (R core Team 2017) (Harrell, 2015). We specified a non–linear regression type model with one imputation and 3 knots for all our datasets which allowed for extrapolation from our data. To determine the appropriate number of knots for our data, we ran a series of imputations which varied in the number of knots and chose the number based on the lowest mean and absolute error (Harrell, 2015). After the imputation for each dataset, a R^2^ value was generated to predict our original measured data from the imputation as a measure of error. High R^2^ values indicate strong predictions. Our R^2^ values for the imputations in all 3 datasets were between 0.37 and 0.59.

**Supplementary Table 2.** Bootstrapping was used to impute missing data to account for differences in sampling effort. Data was bootstrapped to meet the sample size of the largest plant–caterpillar species pairs from our immune assays. Here this is a sample size of 19.

**Supplementary Table 3.** SEM results of bootstrapped data from Costa Rica *Eois* data. We generated 7 a priori hypotheses to explain the relationships between our variables based on previous work on *Piper*. Our hypotheses tested for: I) ‘Herbivore mediation hypothesis’, II) ‘Diet breadth regulation hypothesis’, III) ‘Phytochemical diversity regulation hypothesis’, IV) ‘Combination hypothesis’, V) ‘Interaction hypothesis’, VI) ‘Simple phytochemical diversity hypothesis’, and VII) ‘Immunity does not predict parasitism hypothesis’. Asterisks next to path coefficients indicate statistically significant paths (*P* < 0.05). *Quadrus cerealis* data for VI) ‘Simple phytochemical diversity hypothesis’ did not fit the model. *Eois* data from Ecuador did not fit any of our models.

**
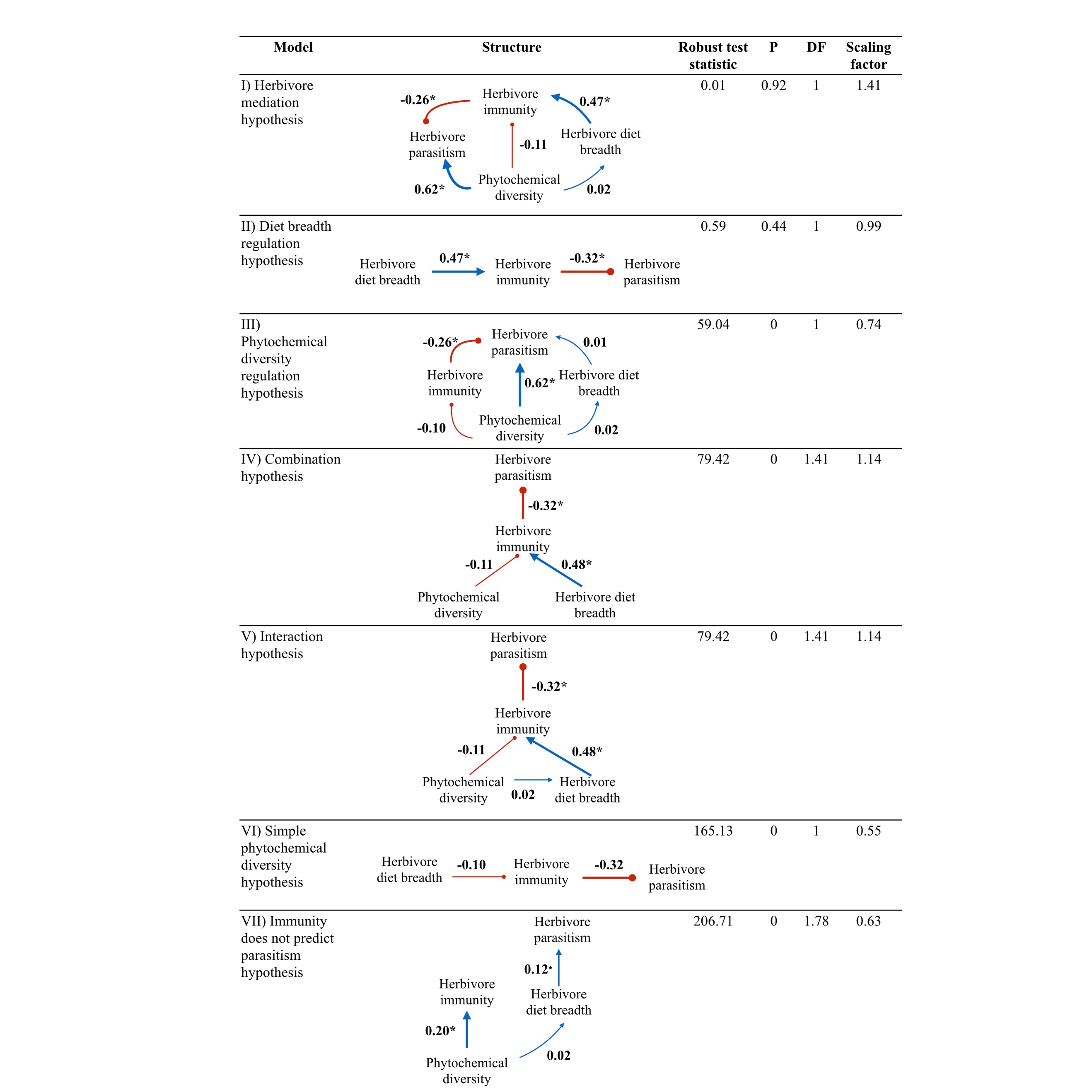
**

**References**

Harrell, F. E. (2015). Package ‘Hmisc’. Retrieved on February 5, 2018.
